# Impact of COVID-19 on TB diagnostic services at primary healthcare clinics in eThekwini district, South Africa

**DOI:** 10.1038/s41598-023-43589-7

**Published:** 2023-10-03

**Authors:** Thobeka Dlangalala, Alfred Musekiwa, Tivani Mashamba-Thompson

**Affiliations:** 1 School of Health Systems and Public Health, Faculty of Health Sciences, University of Pretoria, Pretoria, 0002 South Africa; 2 Faculty of Health Sciences, University of Pretoria, Pretoria, 0002 South Africa

**Keywords:** Health services, Public health, Tuberculosis

## Abstract

We assessed the impact of the pandemic on TB diagnostics at primary healthcare clinics (PHCs) during the different stages of COVID-19 in eThekwini district, South Africa. Data from the District Health Information System (DHIS) were used to conduct an interrupted time series analysis that assessed the changes in TB investigations and confirmed TB cases during four pandemic periods: lockdown and the subsequent three peaks of infection compared to the two years prior (2018–2022). The initial lockdown resulted in − 45% (95% CI − 55 to − 31) and − 40% (95% CI − 59 to − 28) immediate declines in TB investigations and confirmed cases, respectively. Both indicators showed substantial recovery in the months after the first wave (p < 0.05). However, while TB investigations sustained smaller declines throughout the pandemic, they rebounded and surpassed pre-COVID-19 levels by the end of the investigation period. On the other hand, confirmed cases experienced reductions that persisted until the end of the investigation period. TB diagnostic services at PHCs were considerably disrupted by COVID-19, with the confirmation of cases being the most adversely affected throughout the pandemic. The reasons for these persistent declines in TB detection must be determined to inform the development of sustainable diagnostic systems that are capable of withstanding future pandemics.

## Introduction

The outbreak of SARS-CoV-2 brought the world to a standstill in early 2020 due to a lack of effective pharmaceutical interventions which prompted world leaders to implement non-pharmaceutical measures to manage transmission^1^. These actions profoundly impacted the utilization and provision of healthcare services around the globe^2–7^. In addition to population-wide lockdowns, countries repurposed many of their health facilities and human resources to tackle COVID-19^8–10^. This has had a devastating impact on managing other diseases of importance, particularly Tuberculosis (TB) which was disproportionately affected^11^.

TB prevention and care were severely impacted by the wake of the COVID-19 pandemic^3,12^. This comes after achieving significant milestones in reducing the global burden^12^. The TB area most affected by the pandemic was detection, specifically the number of newly diagnosed cases which fell by 18% in 2020 and have only slightly recovered in the year 2021^12^. Though, not ideal these outcomes are not surprising since many diagnostic platforms were repurposed for COVID-19^13^, testing facilities shut down^14,15^, and given the similarities in the clinical presentations of COVID-19 and TB^16^, fear and stigma prevented patients from seeking care^3^. All these resulted in patients presenting later to health facilities thus reducing TB testing in 2020^17^. Consequently, this reduction in TB detection particularly in high-burden countries has increased TB incidence by 3.6% between 2020 and 2021^12^.

South Africa, faces a dual burden of TB and HIV with an estimated 60% of new cases coming from those living with HIV^18^. Given the deadly nature of opportunistic TB infection among people living with HIV, a reduction in TB detection will have dire consequences for the country. Nevertheless, South African TB diagnostic services suffered a similar fate to the rest of the world as a consequence of COVID-19^13,19,20^. During the first lockdown, TB tests conducted using the Xpert MTB/RIF Ultra assay, the primary diagnostic tool in South Africa, decreased by 33%^21^. Between January 2020 and February 2021 the number of people screened for TB and positive TB tests decreased by 19.2% and 18%, respectively, compared to pre-pandemic years^19^. A study from the Limpopo province recorded a 33% reduction in the number of positive TB tests during the initial lockdown^20^. Therefore, finding the missed TB cases and rebuilding stronger TB diagnostic services should be a top priority for the country.

Much of the published literature on TB detection in South Africa is from the start of the pandemic^13,19,22^. Little is known about the state of TB detection in the country’s high-burden settings, particularly, during the different waves of COVID-19 infection. Therefore, this study aimed to estimate the impact of COVID-19 on TB detection at different stages of the pandemic (March 2020–June 2022) compared to two pre-pandemic years (2018–2019) using TB investigations and confirmation of TB cases as indicators. This was done using data from Primary Healthcare Clinics (PHCs) in eThekwini a high TB burden region in South Africa. To our knowledge, a study of this nature has not been conducted in South Africa and the findings can be used to implement targeted interventions for building stronger health systems.

## Methods

This study was conducted as part of a larger research project assessing the effects of COVID-19 on TB diagnostics services at PHCs with the aim of optimizing these services for health crises such as COVID-19^23^.

### Study setting

The study was conducted in the eThekwini district of the KwaZulu-Natal (KZN) province. The district boasts a population of approximately 3.9 million people making it the most populated region in KZN^24^. TB and HIV account for the highest number of deaths by communicable infection within eThekwini, especially among men^24^. Healthcare is provided through a mixture of PHCs, regional hospitals, and one provincial and central hospital^24^. However, TB diagnosis is mainly achieved through passive case finding at PHCs^24,25^. eThekwini has been the epicenter of the COVID-19 pandemic within the province and has recorded 358 222 active cases and 5 707 deaths as of 1 February 2023, respectively^26^.

In South Africa, the first case of COVID-19 was diagnosed on the 5th of March 2020^27^. By the 15th of March 2020, the government declared a national state of disaster in response to the COVID-19 pandemic^28^. With the increasing number of cases, the first lockdown was implemented at midnight on the 26th of March 2020^19^. The peak of infection for the first wave of COVID-19 was reached in July 2020, and the peaks for the second, third, and fourth waves were reached in January 2021, July 2021, and December 2021, respectively^29^. Following the decrease in COVID-19 cases and the increase in population immunity, the South African government terminated the national state of disaster on 5 April 2022^28^.

### Data sources

South Africa collects and records routine data from primary healthcare facilities in the District of Health Information System (DHIS)^30^. Aggregated monthly data on TB investigations and confirmed TB cases from PHCs in eThekwini were extracted from the DHIS. TB investigations entail all inquiries into TB symptoms while the confirmation of TB represents a positive diagnosis following a TB investigation (new cases and relapses). Clinics with missing data points were excluded from the analysis.

### Outcomes

The outcomes of interest were expressed as the total number of TB investigations and the confirmed number of TB cases at PHCs, taken in monthly intervals. After excluding clinics with incomplete data sets and those with outlier values, 94 and 76 facilities were used to analyze TB investigations and confirmation of TB, respectively. The predictions of the outcome variables will be represented by a line graph against a scatterplot of the actual values over time.

### Study design

A single group interrupted time series (ITS) analysis was conducted^31^ using the STATA statistical software version 15.1. The analysis determined whether the exposure (COVID-19) had an immediate or long-term impact on TB investigations and confirmations of TB at PHCs. A times series is the strongest quasi-experimental design that assesses time-delimited exposures such as COVID-19 on selected outcomes^32^. As such, multiple exposure periods throughout the pandemic were investigated which corresponded to different surges of COVID-19 infection in South Africa. Exposure period 1—April 2020, the first month of the level five lockdown; Exposure period 2—July 2020, the peak of the second wave of infection; Exposure period 3—January 2021, the peak of the third wave of infection; Exposure period 4—July 2021, the peak of the second wave of infection; Exposure period 5—December 2021, the peak of the third wave of infection. The outcomes were compared to the two years before the pandemic started (January 2018- February 2020).

### Statistical analysis

The statistical analysis was conducted in Stata version 15^33^. The analysis assumped that a linear relationship existed between time and the respective outcome variables within each segment. Specifically, a least squares regression line was fitted to each segment of the time variable^32^. The model was able to determine the impact of the respective exposures on both outcomes, immediately and over time. The model used terms to investigate the following variables: a constant representing the respective outcome level at the baseline, before COVID-19, and terms describing the immediate changes to outcome levels following a respective COVID-19 exposure as well as the changes in monthly trend after the exposure. The percent change for both indicators immediately after a respective exposure was also reported. The analysis used follows an Ordinary Least Squares (OLS) regression model which assumes that the error terms at respective observations are uncorrelated. Thus, to fit a model that accounts for autocorrelation the Cumby–Huizinga general test^34^ was used to assess autocorrelation. The test plotted up to lag order = 12 to assess autocorrelation and seasonality. Newey-west standard errors accounted for autocorrelation. The detailed regression model used for this study is outlined in Supplementary File 1.

### Ethics declaration

The study was conducted according to the Helsinki Declaration and the South African POPIA Act^35^. Before the commencement of the analyses, ethical approval was sought and granted by the University of Pretoria’s Faculty of Health Sciences Research Ethics Committee (reference number: 652/2021) and from the Health Research and Management Unit of the KZN Department of Health (NHRD Ref: KZ_202112_012). No human participants were involved in this study, therefore, the need for informed consent was waived by the University of Pretoria’s Faculty of Health Sciences Research Ethics Committee.

## Results

### Characteristics of healthcare facilities

A total of 94 healthcare facilities were included in the analyses of TB investigations and 74 for TB confirmations, respectively (Table 1). These facilities comprised primary healthcare clinics (87% and 86%), community health centers (9%), gateway (3% and 4%), and a polyclinic (1%) for investigations and confirmations, respectively. An average of 9965 TB investigations were conducted from health facilities and 754 cases were confirmed as TB every month.

**Table 1:** Characteristics of the included primary healthcare facilities.

Facility type	TB investigations			TB confirmations		
	No. present	%	Mean monthly Investigations	No. present	%	Mean monthly confirmations
Community health centers	7	9	1520 ± 128.5	7	9	201 ± 18.6
Gateway clinics	3	3	337 ± 74.2	3	4	40 ± 7.8
Polyclinics	1	1	284 ± 0.0	1	1	52 ± 0.0
Primary healthcare clinics	83	87	7824 ± 91.2	65	86	463 ± 6.2
Total	94	100	9965	76	100	754

**Figure 1:**
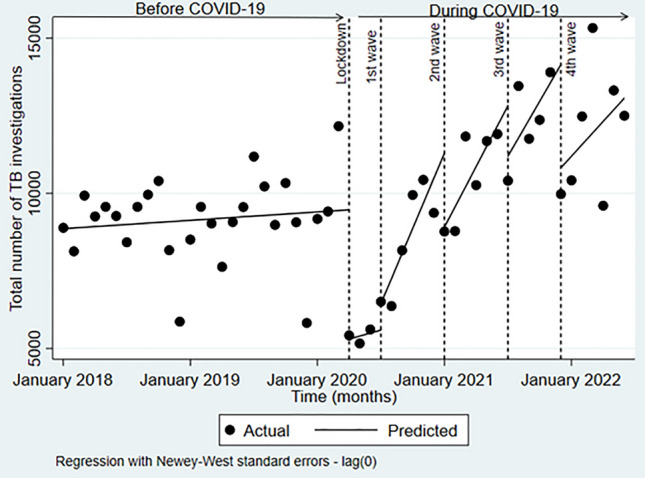
Impact of COVID-19 on TB investigations at PHCs in the eThekwini district, South Africa (January 2018–June 2022). Dashed vertical lines represent the April lockdown and the peaks of the four waves of COVID-19. No autocorrelation was present in the dataset.

**Figure 2:**
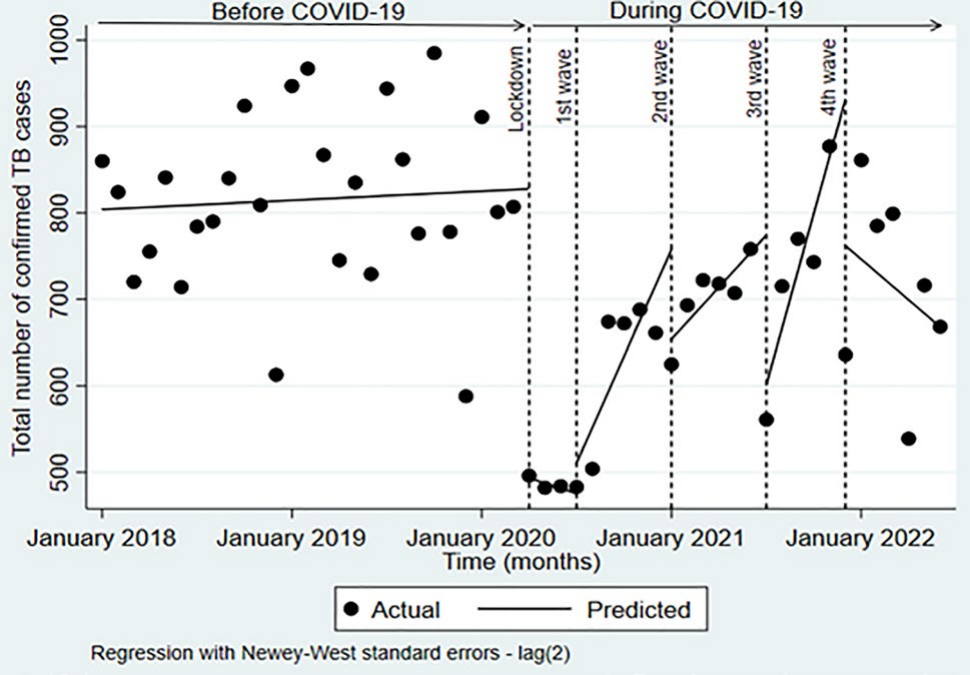
Impact of COVID-19 on TB confirmations at PHCs in the eThekwini district, South Africa (January 2018–June 2022). Dashed vertical lines represent the April lockdown and the peaks of the four waves of COVID-19. Autocorrelation up to lag 2 was adjusted for using Newey-West standard errors.

The onset of the pandemic resulted in TB investigations and confirmations being reduced by approximately half (Table 2). TB investigations increased with each subsequent wave reaching pre-pandemic levels by the fourth wave, however, confirmation of cases did not rebound in this manner. The segmented linear regression analysis was used to test the statistical significance of the immediate and long-term effects of the pandemic (lockdown and subsequent waves) on TB investigations and TB confirmations (Figs. 1 and 2). COVID-19 significantly impacted both TB indicators, although, the impact was heterogeneous across the different stages of the pandemic.

**Table 2:** Monthly changes in TB investigations and confirmations at PHCs, before COVID-19 and at different stages of the pandemic.

	Period	Number of TB investigations	Number of TB confirmations
Before COVID-19; monthly mean	Jan–Dec 2018	8950	790
	Jan–Dec 2019	9080	835
	Jan–Mar 2020	10,251	840
During COVID-19; actual monthly value	Lockdown Apr 2020	5521	496
	1st wave Jul 2020	6507	483
	2nd wave Jan 2021	8766	625
	3rd wave Jul 2021	10,406	561
	4th wave Dec 2021	9973	636

### TB investigations

Before the COVID-19 pandemic, TB investigations at PHCs were increasing slightly by approximately 23 tests per month from a baseline of 8 860 (95% CI 7998 to 9722) (Fig. 1; Table 3). After the emergence of COVID-19 and the implementation of the level 5 lockdown, the number of investigations declined by 4 165 (95% CI − 5669 to − 2660) which represented a − 45% (95% CI − 55 to − 31) decline relative to the counterfactual. The trends in monthly tests following the lockdown did not differ substantially from those experienced before the pandemic.

**Table 3:** Statistical changes in TB diagnostic indicators during different phases of COVID-19 (April 2020 Lockdown–January 2021).

	TB investigations	TB confirmations
Before COVID-19		
Starting level intercept	8860	803
Slope before intervention (95% CI)	23 (− 55 to 100)	1 (CI − 3 to 5)
* p*-value	0.56	0.617
Lockdown April 2020		
Change in the month of exposure (95% CI)	− 4165 (− 5669 to − 2 660)	− 334 (− 398 to − 271)
Percent change (95% CI)	− 45% (− 55 to − 31)	− 40% (− 59 to − 28)
Average monthly change (95% CI)	74 (− 133 to 280)	− 7 (95% CI − 12 to − 2)
* p*-value	0.48	0.006
1st wave July 2020		
Change in the month of exposure	852 (− 196 to 1899)	35 (− 33 to 104)
Percent change (95% CI)	16 (− 4 to 34)	7 (− 10 to 15)
Average monthly change (95%CI)	712 (234 to 1189)	47 (27 to 68)
* p*-value	0.004	≤ 0.0001
2nd wave Jan 2021		
Change in the month of exposure (95% CI)	− 2385 (− 4701 to − 70)	− 104 (− 183 to − 25)
Percent change (95% CI)	− 23 (− 32 to − 1)	− 12 (− 20 to − 3)
Average monthly change (95% CI)	− 156 (− 643 to 332)	21 (− 48 to 5)
* p*-value	0.523	0.118
3rd wave July 2021		
Change in the month of exposure (95% CI)	− 1627 (− 3647 to 392)	− 173 (− 222 to − 124)
Percent change (95% CI)	− 22 (− 63 to 4)	− 29 (− 52 to − 6)
Average monthly change (95% CI)	− 63 (− 669 to 543)	46 (23 to 68)
* p*-value	0.84	≤ 0.0001
4th wave December 2021		
Change in the month of exposure (95% CI)	− 3326 (− 5435 to − 1218)	− 169 (− 293 to − 46)
Percent change (95% CI)	− 33 (− 51 to − 15)	− 26 (− 92 to − 4)
Average monthly change	− 215 (− 897 to 467)	− 82 (− 117 to − 47)
* p*-value	0.528	≤ 0.001

The table shows the estimates for the absolute changes in the month of the exposure and the monthly trends thereafter for TB investigations and confirmation of cases during the pre-pandemic period, the lockdown, and the subsequent four waves of infection that followed.

During the peak of the first wave in July 2020, TB investigations showed a relative increase of 16% (95% CI − 4 to 34) (n = 852; 95% CI − 196 to 1899) compared to the lockdown period. However, the trends in investigations after the first wave increased significantly month-to-month by 712 (95% CI 235 to 1189) compared to the period prior p = 0.04.

After five months of marked increases the peak of the second wave, in January of 2021, brought immediate declines to TB investigations of − 23% (95% CI − 32 to − 2) (n = − 156; 95% CI − 643 to 332). The declines persisted month-to-month after the peak though not significantly (p > 0.05).

By the third peak of the third wave which was driven by the emergence of the delta variant, the level and subsequent monthly trends of TB investigations continued to decline but in a non-significant manner compared to the 2nd wave.

When the fourth wave peaked, in December 2021 the level of TB investigations dropped by -33% (95% CI 51 to 15) (n = − 3326; 95% CI: − 5435 to − 1218) compared to the 3rd wave. The monthly trends after the peak showed negligible decreases, however, the graphical representation shows that overall, more TB investigations were being conducted in this period than in the time before the COVID-19 pandemic (Fig. 1).

### TB confirmations

The monthly trend in the confirmation of cases remained stable before COVID-19, showing little deviation from the baseline amount of 803 (Fig. 2; Table 3). The inception of the pandemic resulted in TB confirmations dropping significantly by − 334 (95% CI − 398 to − 271) in the month of the lockdown which corresponded to − 40% (95% CI 59 to − 28) decline compared with the counterfactual. The significant declines continued month-to-month by approximately 7 cases (95% CI − 11 to − 2) after the lockdown (Fig. 2; Table 3).

At the peak of the 1st wave, the level of TB confirmations rose slightly by 7% (95% CI − 10 to 15) (n = 35; 95% CI − 33 to 104) relative to the lockdown period. In contrast, the monthly trends following the first wave resulted in statistically significant increases of approximately 47 (95% CI − 33 to 104) compared to the previous period (p ≤ 0.0001).

During the peak of the second wave, the level of TB confirmations experienced a drop of approximately − 12% (95% CI − 20 to − 3) (n = − 104; 95% CI − 183 to − 25). The declines persisted through the months that followed though not significantly.

When the third wave peaked, confirmed cases of TB dropped instantaneously by approximately − 29% (95% CI − 52 to − 6) (n = − 173; 95% CI − 222 to − 124). However, they rose again by an average of 46 (95% CI 23 to 68) monthly cases after the peak compared to the previous wave.

At the peak of the fourth wave, TB confirmations reduced once more by − 26% (95% CI − 92 to − 4) (n = − 169; 95% CI − 293 to − 46) compared to the prior period. The significant declines continued in the months after the peak reducing to monthly estimates lower than those observed before the pandemic (Fig. 2).

## Discussion

The regression analysis showed that TB investigation and confirmations of cases at PHCs in eThekwini were greatly affected by COVID-19 but to varying degrees. The largest declines for both indicators occurred during the level-five lockdown period. Conversely, the 1st peak of infection and the months that followed were marked by substantial increases in the monthly trends for both indicators. The following waves of infection had heterogenous effects on the indicators though generally marked by substantial declines at the peaks. Lastly, the confirmation of diagnosis was more severely impacted by the pandemic recording decreases that persisted until the end of the observation period.

Our results reported drastic declines of 40% and 45% for both TB detection indicators at the beginning of the pandemic. These were similar to the reduction that was reported in Uganda (43%)^36^ and slightly lower than the reductions experienced by Malawi (39.5%)^10^, Nigeria (34%)^5^, Brazil (26.4%)^37^ and Zambia (22%)^7^. The reasons for the declines were similar, namely, mitigation measures placed restrictions on non-essential travel, and fear of contracting the virus was also heightened during this period which limited the use of health facilities. In China and certain SSA countries the TB case notifications began to rebound in the months to follow^5,7–10^, Malawi and Zambia even recording pre-pandemic numbers by December of 2020 and September 2021, respectively^7,10^. These increases were either a result of efforts by respective governments to scale up TB response activities or the relaxing of COVID-19 related restrictions. Similarly, our study also found that both TB indicators began to rebound substantially after the level five lockdown was lifted even returning to pre-pandemic levels by September 2020 when the country moved to level 1 lockdown. This demonstrates the importance of maintaining ease of access to healthcare services during a health crisis and the need for corrective strategies in areas where access was compromised.

Though there has been extensive documentation of the pandemic's effects on TB detection^5–10,20^. These studies are limited to the lockdown period and very few studies have considered the longitudinal impact of the pandemic on important TB service indicators^7,20^. As such, many studies have drawn premature conclusions based on the information available at the time. For instance, a South African study showing the rapid improvement in TB testing after the initial lockdown hypothesized that the quick recovery would not negatively impact TB incidence in the future^22^. Similarly, the World Health Organization has reported that TB case detection in South Africa during 2020 and 2021 was similar to pre-pandemic levels^12^. However, our study reveals that in eThekwini diagnostic services, particularly the confirmation of cases encountered significant declines throughout most of the pandemic suggesting that specific regions within the country were disproportionately affected. We expected confirmation of cases to mirror the pattern of TB investigations given their association with each other. This unexpected reduction in confirmed diagnoses may be due to underreporting or underdiagnosis. Either possibility would have negative implications for disease surveillance and transmission, respectively.

Studies are required to understand what caused the continued reduction of TB detection at PHCs during COVID-19 in eThekwini. Since the effect of undetected TB on mortality is more severe and noticeable in the short term^12^, this metric should be monitored to determine whether a true reduction in diagnosed TB took place instead of an increase in underreporting. Given that reduction in diagnoses was more exacerbated during peaks in COVID-19, TB testing capacity at central laboratories may have been compromised at this time because Gene Xpert MTB/RIF machines were repurposed for COVID-19^13^. Likewise, staff and resources at PHCs may have been overstretched at the time of COVID-19 surges leading to underreporting at facility level. Both these hypotheses would need to be confirmed with qualitative studies at both PHCs and laboratories. Health systems should be capable of providing quality care and positive health outcomes despite ongoing health crises^38^. Failure to learn from the pandemic and produce resilient health systems in addition to identifying the cases that may have been missed during the pandemic will have dire consequences for TB management in South Africa.

The study was not without limitations, firstly the data used was from the DHIS and therefore subject to human error during the manual capturing. It is also possible that errors and missing data may have been amplified by the stresses brought on by the pandemic. To help mitigate data inaccuracies, clinics with missing data and outliers were excluded from the analyses. Another limitation was the use of a single-group ITS analysis which by design lacks a control group and assumes that any existing confounders are changing relatively slowly over time such that they would not interfere with the analysis. Including a comparable control group was not possible because COVID-19 impacted the entire country. However, it is unlikely that factors other than the pandemic caused the observed effects on the outcomes given the abrupt and drastic changes that occurred simultaneously with the various stages of COVID-19. Furthermore, the study only investigated the impact of COVID-19 on TB diagnosis and did not consider the development of drug resistance or TB outcomes which were also affected by the pandemic.

Some strengths of this study were the use of a time series analysis, which is the best quasi-experimental design for estimating the effects of an exposure on an outcome when randomization is not possible^32^. Secondly, using multiple exposure periods provides a robust illustration of the impact of COVID-19 on TB detection.

By assessing the patterns of TB diagnostic services at PHCs before and throughout COVID-19 we were able to determine the longitudinal impact of the pandemic on these services. Both TB investigations and confirmed cases of TB were negatively impacted by the level five lockdown, however, confirmed TB remained on the decline despite investigations rebounding as the pandemic continued. The causes for the reductions remain unclear and need to be investigated to strengthen diagnostics at PHCs both currently and for future pandemics.

### Supplementary Information

Supplementary Information.

## Data Availability

The datasets generated during and/or analyzed during the current study are available from the corresponding author upon reasonable request.

## Abbreviations

DHIS: District of health information system
ITS: Interrupted time series
KZN: KwaZulu-Natal
PHCs: Primary healthcare clinics
SSA: Sub-Saharan Africa
TB: Tuberculosis

## References

[1] Anderson RM, Heesterbeek H, Klinkenberg D, Hollingsworth TD (2020). How will country-based mitigation measures influence the course of the COVID-19 epidemic?. Lancet.

[2] Xiao H (2021). The impact of the COVID-19 pandemic on health services utilization in China: Time-series analyses for 2016–2020. Lancet Reg. Health Western Pac..

[3] Dlangalala T (2021). Evidence of TB services at primary healthcare level during COVID-19: A scoping review. Diagnostics.

[4] Siedner MJ (2020). Access to primary healthcare during lockdown measures for COVID-19 in rural South Africa: A longitudinal cohort study. MedRxiv.

[5] Adewole OO (2020). Impact of COVID-19 on TB care: Experiences of a treatment centre in Nigeria. Int. J. Tuberc. Lung. Dis..

[6] Rai DK, Kumar R, Pandey SK (2020). Problems faced by tuberculosis patients during COVID-19 pandemic: Urgent need to intervene. Indian J. Tuberc..

[7] Lungu P (2022). Interrupted time-series analysis of active case-finding for tuberculosis during the COVID-19 pandemic, Zambia. Bull. World Health Org..

[8] Fatima R, Akhtar N, Yaqoob A, Harries AD, Khan MS (2021). Building better tuberculosis control systems in a post-COVID world: Learning from Pakistan during the COVID-19 pandemic. Int. J. Infect. Dis..

[9] Chen H, Zhang K (2020). Insight into impact of COVID-19 epidemic on tuberculosis burden in China. Eur. Respir. J..

[10] Soko RN (2021). Effects of coronavirus disease pandemic on tuberculosis notifications, Malawi. Emerg. Infect. Dis..

[11] World Health Organization. *Global Tuberculosis Report*. https://www.who.int/teams/global-tuberculosis-programme/tb-reports (2021).

[12] World Health Organization. *Global Tuberculosis Report*. https://www.who.int/teams/global-tuberculosis-programme/tb-reports/global-tuberculosis-report-2022 (2022).

[13] Karim QA, Karim SSA (2020). COVID-19 affects HIV and tuberculosis care. Science.

[14] Burzynski JJ, Macaraig M, Nilsen D, Schluger NW (2020). Transforming essential services for tuberculosis during the COVID-19 pandemic: Lessons from New York City. Int. J. Tuberc. Lung Dis..

[15] Apolisi I (2022). Supporting families with tuberculosis during COVID-19 in Khayelithsa, South Africa. Lancet Respir. Med..

[16] Alene KA, Wangdi K, Clements ACA (2020). Impact of the COVID-19 pandemic on tuberculosis control: An overview. Trop. Med. Infect. Dis..

[17] Aznar ML (2021). Impact of the COVID-19 pandemic on tuberculosis management in Spain. Int. J. Infect. Dis..

[18] UNAIDS. *90–90–90 Treatment for ALL GENEVA: UNAIDS*, https://www.unaids.org/en/resources/909090 (2020).

[19] Pillay Y, Pienaar S, Barron P, Zondi T (2021). Impact of COVID-19 on routine primary healthcare services in South Africa. S. Afr. Med. J..

[20] Mutyambizi C (2021). Effect of COVID-19 on HIV, tuberculosis, and prevention of mother-to-child transmission of HIV indicators in Mopani district, South Africa. S. Afr. Med. J..

[21] National Institute for Communicable Diseases. *Impact of COVID-19 Intervention on TB Testing in South Africa*. https://www.nicd.ac.za/wp-content/uploads/2020/05/Impact-of-Covid-19-interventions-on-TB-testing-in-South-Africa-10-May-2020.pdf (2020).

[22] Abdool Karim Q, Baxter C (2022). COVID-19: Impact on the HIV and tuberculosis response, service delivery, and research in South Africa. Curr. HIV/AIDS Rep..

[23] Dlangalala T, Musekiwa A, Mashamba-Thompson T (2022). Towards development of a novel approach for enhancement of TB diagnostic services during the pandemic: A case of primary health care clinics in eThekwini district KwaZulu-Natal: A study protocol. PLOS ONE.

[24] District Development Model. *About EThekwini*. https://www.durban.gov.za/pages/government/about-ethekwini (2020).

[25] Osman M (2021). Health system determinants of tuberculosis mortality in South Africa: A causal loop model. BMC Health Serv. Res..

[26] KwaZulu-Natal Department of Health. *COVID-19*. http://www.kznhealth.gov.za/coronavirus.htm (2023).

[27] National Institute for Communicable Diseases. *First Case of COVID-19 Coronavirus Reported in SA | NICD*. https://www.nicd.ac.za/first-case-of-covid-19-coronavirus-reported-in-sa/ (2020).

[28] Nicholson, G. *Daily Maverick* (South Africa, 2022).

[29] Worldometer. *Total Coronavirus Cases in South Africa*. https://www.worldometers.info/coronavirus/country/south-africa/ (2022).

[30] Shaw V (2005). Health information system reform in South Africa: Developing an essential data set. Bull. World Health Organ..

[31] Linden A (2015). Conducting interrupted time-series analysis for single- and multiple-group comparisons. Stata J..

[32] Wagner AK, Soumerai SB, Zhang F, Ross-Degnan D (2002). Segmented regression analysis of interrupted time series studies in medication use research. J. Clin. Pharm. Ther..

[33] StataCorp. *Stata Statistical Software: Release 15*. (StataCorp LLC, 2017).

[34] Cumby RE, Huizinga J (1992). Testing the autocorrelation structure of disturbances in ordinary least squares and instrumental variables regressions. Econometrica.

[35] Adams R, Adeleke F, Anderson D, Bawa A, Branson N, Christoffels A (2021). POPIA Code of Conduct for Research. S. Afr. J Sci..

[36] Kadota JL (2020). Impact of shelter-in-place on TB case notifications and mortality during the COVID-19 pandemic. Int. J. Tuberc. Lung Dis..

[37] de Souza CDF, Coutinho HS, Costa MM, Magalhães M, Carmo RF (2020). Impact of COVID-19 on TB diagnosis in Northeastern Brazil. Int. J. Tuberc. Lung Dis..

[38] Kruk ME, Myers M, Varpilah ST, Dahn BT (2015). What is a resilient health system? Lessons from Ebola. Lancet.

