# Supplementary material for: Impact of COVID-19 on TB diagnostic services at primary healthcare clinics in eThekwini district, South Africa
Source: Sci Rep. 2023 Oct 3;13:16645. doi: 10.1038/s41598-023-43589-7 (PMC10547754; doi:10.1038/s41598-023-43589-7)
Supplement: Supplementary file 1 — Supplementary Information. [file 41598_2023_43589_MOESM1_ESM.pdf]

Impact of COVID-19 on TB diagnostic services at primary healthcare clinics in eThekwini district, South Africa

Thobeka Dlangalala[1]*, Alfred Musekiwa[1], Tivani Mashamba-Thompson[3]

Statistical Analysis

To estimate the impact of the exposure on the level and trend of the outcome variables the following regression model was used:

$$Y_{t=}\ \beta_0 + \beta_1 T_t + \beta_2 Exposure1_t + \beta_3 Time\ after\ Exposure1_t + \beta_4 Exposure2_t + \beta_5 Time\ after\ Exposure2_t + \beta_6 Exposure3_t + \beta_7 Time\ after\ Exposure3_t + \beta_8 Exposure4_t + \beta_9 Time\ after\ Exposure4_t + \beta_{10} Exposure5_t + \beta_{11} Time\ after\ Exposure5_t + \varepsilon_t$$

Y_t represents the outcomes, either TB investigations or confirmation of TB cases measured per month *t*, respectively. *Tt* represents the time, in months, that has elapsed since the beginning of the series. $Exposure_t$ is a dummy variable that is equal to 0 in the pre-exposure and 1 in the post-exposure period. $Time\ after\ Exposure_t$ is the interaction factor between a specified exposure and time. β_0 is the starting level of the outcomes at time zero, respectively. The coefficient β_1 measures the monthly change in the outcomes in the pre-pandemic period while $\beta_{2,4,6,8,10}$ denotes the changes in the outcome levels immediately after a respective exposure period. $B_{3,5,7,9,11}$ represents the changes in the slope of the outcomes pre- and post-exposure period of interest. P-value ≤ 0.05 will be used as the measure of statistical significance. Thus, significant *p*-values in $\beta_{2,4,6,8,10}$ will show an immediate exposure effect whereas a significant *p*-value in $\beta_{3,5,7,9,11}$ represents a change over time.[1] ε_t is the error term for any arising random variability that has not been accounted for by the model.

1 Linden, A. Conducting Interrupted Time-series Analysis for Single- and Multiple-group Comparisons. *The Stata Journal* **15**, 480-500, doi:10.1177/1536867x1501500208 (2015).

2 Cumby, R. E. & Huizinga, J. Testing the Autocorrelation Structure of Disturbances in Ordinary Least Squares and Instrumental Variables Regressions. *Econometrica* **60**, 185, doi:10.2307/2951684 (1992).
